# Vital Signs in Palliative Care: A Scoping Review

**DOI:** 10.3390/cancers15184641

**Published:** 2023-09-20

**Authors:** Jenny Power, Craig Gouldthorpe, Andrew Davies

**Affiliations:** 1 School of Medicine, University College Dublin, D04 V1W8 Dublin, Ireland; 2 Our Lady’s Hospice & Care Services, D6W RY72 Dublin, Ireland; 3 School of Medicine, Trinity College Dublin, D02 PN40 Dublin, Ireland

**Keywords:** cancer, palliative care, vital signs, prognostication

## Abstract

**Simple Summary:**

Vital signs (heart rate, blood pressure, respiratory rate, oxygen saturation, and temperature) are often used in healthcare settings as indicators of how unwell a patient is, and how they are responding to treatment. They are often not measured routinely for patients who are receiving palliative care. This review highlights that measuring vital signs for patients with cancer who are receiving palliative care may be of some benefit in determining prognosis, but further studies are needed to clarify the extent of association between a patient’s vital signs and prognosis.

**Abstract:**

Vital signs are routinely measured in secondary healthcare settings and can be used to detect clinical problems, guide treatment, and monitor response to treatment. Vital signs are less frequently measured in palliative care settings. Reasons for this are unclear. This scoping review aimed to assess the generic use of vital signs in palliative care, and its role in prognostication for adult patients with cancer receiving palliative care. Medline, Embase, and CINAHL were searched for articles involving adult patients with advanced cancer receiving palliative care who had their vital signs measured. Twenty-six articles were identified in which one or a combination of vital signs, with or without other clinical parameters, was used to prognosticate for patients. An additional three articles investigated the generic use of vital signs in patients with advanced cancer. There was significant heterogeneity between identified studies, with some indication that changes in vital signs may indicate that a patient is close to death. However, other studies suggested that patients may maintain normal vital signs until the time of death. Further studies are needed to explore whether abnormal vital signs may be used as a prognostic indicator for patients with cancer receiving palliative care.

## 1. Introduction

The Oxford English Dictionary defines a vital sign as “a clinical measurement that indicates the state of a patient’s essential body functions” [1]. The four “primary” vital signs are respiratory rate, heart (pulse) rate, blood pressure, and temperature. However, numerous other parameters have also been suggested as vital signs, e.g., oxygen saturation [2], blood glucose level, skin (appearance, temperature), and pupil (size, reactivity). Pain has been previously described as the “fifth vital sign” [3], but vital signs are always objective, and pain intensity/severity is always subjective.

Vital signs are routinely measured in secondary healthcare settings, and form the basis of many so-called “early warning systems” (to detect significant clinical problems) [4]. In addition to detecting clinical problems, they can monitor the response to initial treatment (and so guide ongoing treatment). Furthermore, “abnormal” vital signs have been shown to be prognostic indicators in hospital inpatients [5]. Vital signs are regularly utilised in other (i.e., non-secondary) healthcare settings, although they are less frequently utilised in palliative care settings (e.g., hospices, homecare services) [6].

It remains unclear why there is this disparity in the measurement of vital signs, although possible reasons include perceived patient burden (general disturbance, physical discomfort), perceived clinical futility [7], and the “medicalisation of death” [8]. However, today, many patients receiving palliative care are also receiving disease-modifying treatment, and have an expected prognosis of months or longer (and so would be appropriate for “aggressive” management of acute clinical problems). Thus, it is important to undertake a review of the utility of measurement of vital signs in this cohort of patients.

Importantly, vital signs are affected by a number of different (non-disease) factors, and there is an ongoing problem with inter-observer variability in measurement [9]. The normal respiratory rate is 12–20/min, with higher rates in pregnant women and children (especially younger children). Similarly, the normal heart (pulse) rate is 60–100/min, again, with higher rates in children (especially in younger children). The normal upper range for systolic blood pressure is <120 mmHg (with a lower range of 90 mmHg), and the normal upper range for diastolic blood pressure is <80 mmHg (with a lower range of 60 mmHg) [9,10]. Blood pressure readings are affected by a number of factors, including patient actions, patient posture, arm position (and support), and cuff size; for example, talking/active listening adds 10 mmHg to readings, and using a cuff size that is too small leads to falsely high readings (and vice versa) [9]. The normal body temperature is 36.5–37.5 °C, and there is a circadian variation in body temperature (i.e., lowest in the morning, highest in the evening). Tympanic/axillary measurements are less accurate estimations of body temperature than oral/rectal measurements.

The aim of this scoping review was to review the literature on vital signs in palliative care (and specifically in patients with advanced cancer), with the objectives of assessing their generic use, as well as their use as potential prognostic indicators.

## 2. Methods

The methodology utilised in this review was based on the framework developed by Arksey and O’Malley [11], but incorporated updated guidance on this framework [12]. The PRISMA Extension for Scoping Reviews (PRISMA-ScR) was used to report the outcome of this review [13].

### 2.1. Search Strategy

Three electronic databases (Medline, Embase, CINAHL) were originally searched in March 2022, and re-searched in June 2023 (to check for any new references). A detailed search strategy was developed for CINAHL and adapted as needed for the other databases. The search strategy was developed by the lead author, with support from the medical librarian, and reviewed by the two other authors (CG and AD). Non-English studies and abstracts without associated full texts were excluded from the review. The initial search included all diagnoses, and was then restricted to patients with advanced cancer.

A consensus was reached on the vital signs to include in the search strategy; it was determined by a review of the routinely measured vital signs in secondary care settings, and particularly vital signs used in commonplace “early warning scores” [4]. Thus, the vital signs included were respiratory rate, heart rate, blood pressure, temperature, and oxygen saturation.

The search strategy within CINAHL is as follows: vital signs or vital sign monitoring or vital sign assessment OR physiological monitoring OR (MH “Blood Pressure”) OR (MH “Blood Pressure Determination”) OR (MH “Systolic Pressure”) OR (MH “Diastolic Pressure”) OR (MH “Hypertension”) OR hypotension OR (MH “Hypotension”) OR (MH “Plethysmography”) OR “plethysmography” OR “photoplethysmography” OR “remote photoplethysmography” OR (MH “Heart Rate”) OR “heart rate” OR (MH “Tachycardia”) OR “tachycardia” OR (MH “Bradycardia”) OR “bradycardia” OR (MH “Respiratory Rate”) OR “respiratory rate” OR “respiration rate” OR (MH “Tachypnea”) OR “tachypnoea” OR “bradypnea” OR (MH “Body Temperature”) OR (MH “Fever”) OR “fever” OR (MH “Hypothermia”) OR “hypothermia” OR “pyrexia” OR (MH “Oxygen Saturation”) OR “oxygen saturation” OR “spo2” OR “hypoxia” AND (MH “Palliative Care”) OR “palliative care” OR (MH “Palliative Medicine”) OR (MH “Terminal Care”) OR “terminal care” OR “end of life care” OR (MH “Hospice Care”) OR “hospice care” AND (MH “Prognosis”) OR “prognosis” OR (MH “Survival”) OR “survival” OR (MH “Death”) OR “death” OR (MH “Mortality”) OR “mortality”.

### 2.2. Study Eligibility Criteria

Studies needed to include patients with advanced cancer, as defined by the National Cancer Institute/NCI, USA [14]: “Cancer that is unlikely to be cured or controlled with treatment”. Studies which included mixed groups of patients were excluded, unless results for the patients with advanced cancer were separately reported. Studies needed to include details of major vital signs (i.e., respiratory rate, heart rate, blood pressure, temperature, and oxygen saturation). Studies involving children (<19 yr) were excluded. Case reports, review articles, and other records without original information were also excluded.

### 2.3. Data Management and Synthesis

The EndNote 20™ bibliographic software (Clarivate Analytics LLP, USA) was used to store the retrieved articles, whilst the Covidence systematic review software (Veritas Health Innovation, Australia) was used to screen these retrieved articles.

Two reviewers (J.P., C.G.) independently screened the titles and abstracts for full text articles to review. Relevant full text papers were then independently assessed for inclusion by the same two reviewers (J.P., C.G.). A third reviewer (A.D.) was available to resolve potential conflicts. Two reviewers (J.P., C.G.) independently reviewed the full text articles, and extracted the relevant information using a review-specific template. A third reviewer (A.D.) was again available to resolve conflicts.

The reference lists of all retrieved full text articles, relevant chapters in major palliative care textbooks, and relevant sections of major palliative care guidelines were hand-searched for other potential studies.

## 3. Results

### 3.1. Search Results

**Figure 1:**
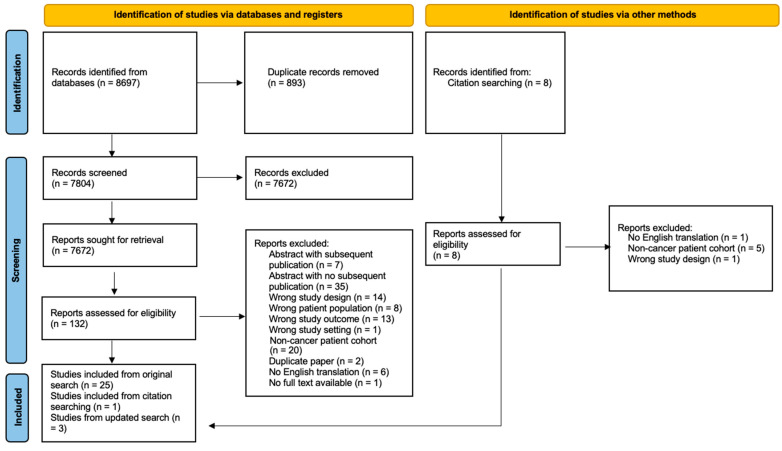
Prisma diagram.

The search strategy identified 8697 references, although only 131 full text articles were retrieved (Figure 1). Twenty-six studies were identified during the initial database searches [15,16,17,18,19,20,21,22,23,24,25,26,27,28,29,30,31,32,33,34,35,36,37,38,39]; three further studies were identified during the re-searches in June 2023 [40,41,42]. The studies identified included 26 prognostication studies [15,16,17,18,19,20,21,22,23,24,25,26,27,28,29,30,31,32,33,34,37,38,39,40,41,43] and three non-prognostication studies [35,36,42]. Table 1 shows data from the prognostication studies (excluding one apparently overlapping study [37], one secondary analysis paper [38], one study involving patients that died after chemotherapy [39], and one study involving patients that died after ICU admission [43]).

### 3.2. Overview of Studies

The prognostication studies involved variable/limited numbers of patients (median: 260; range: 24–3062) [15,16,17,18,19,20,21,22,23,24,25,26,27,28,29,30,31,32,33,34,40,41]. Moreover, the studies assessed different survival durations. Only nine studies assessed all of the “primary” vital signs (i.e., respiratory rate, heart rate, blood pressure, and temperature) [15,20,22,26,28,31,32,40,41], and five of these studies also assessed oxygen saturation [22,26,28,32,41]. Importantly, only six studies involved serial measurements of vital signs [17,24,28,29,32,34], and only three studies involved daily (repeated) measurements of vital signs [28,32,34].

The three non-prognostic studies also involved variable/limited numbers of patients (median: 102; range: 30–798) [35,36,42]. Pearse et al. only assessed blood pressure [35], and this was measured on admission to the hospice. In contrast, Pavic et al. assessed heart rate (and heart rate variability), skin temperature, and oxygen saturation, and these were continuously measured during the study period (up to 12 weeks) [36,44]. Fan et al. assessed heart rate and blood pressure, and this was only assessed on admission to the hospital [42].

### 3.3. Results of Prognostication Studies

Twenty-one of the prognostication studies reported an association between one or more “abnormal” vital sign and overall prognosis: Lam et al. found an association with heart rate (tachycardia) on univariate analysis, but this was not confirmed on multivariate analysis [18]. Respiratory rate (tachypnoea) was a prognostic indicator in eight studies [16,17,19,21,22,29,34,43]; heart rate (tachycardia) was predictive in 16 studies [16,17,20,21,22,23,24,25,26,28,29,30,31,33,34,40]; low systolic blood pressure was predictive in seven studies [15,20,24,25,26,28,40]; low diastolic blood pressure was predictive in three studies [24,28,40]; lower temperature was predictive in three studies [26,27,40]; high temperature was identified in one study [28]; and low oxygen saturation was predictive in in six studies [24,26,28,29,32,41].

Table 1 shows the relevant statistical parameters, including odds ratios, hazard ratios, relative risks, and positive predictive value (with confidence intervals). Odds ratios were the most common parameter reported (60% of studies), and these varied somewhat between studies. For example, Bruera et al., who measured vital signs twice daily, reported an odds ratio of 2.0 [95% CI: 1.1–3.2] for increased heart rate to predict mortality within 3 days [28]. However, Fukui et al., who measured vital signs every minute, reported an odds ratio of OR = 1.031 [95% CI: 1.013–1.120] for increased heart rate to predict mortality over the same period [34].

Nine articles described prognostic models that incorporated vital signs (as well as other clinical/laboratory parameters) [16,19,21,23,26,30,32,38,40]: the included vital signs were respiratory rate (n = 4) [16,19,21,38], heart rate (n = 8) [16,21,23,26,30,32,38,40], blood pressure (n = 3) [26,32,40], temperature (n = 1) [26], and oxygen saturation (n = 1) [26].

### 3.4. Results of Non-Prognostication Studies

Pearse et al. found that low systolic blood pressure, but not postural hypotension, was an independent risk factor for falls in hospice inpatients [35]. Importantly, the rate of falls was relatively common in this cohort of patients (compared with nursing home residents). Pearse et al. suggested screening for hypotension as part of a broader strategy to reduce falls in hospice inpatients.

Pavic et al. performed a feasibility study of a biosensor “wearable” in patients with cancer receiving palliative care; the biosensor assessed heart rate, heart rate variability, skin temperature, and oxygen saturation [36,44]. One of the endpoints for the study was readmission to hospital, and this endpoint was associated with an increase in heart rate and a decrease in heart rate variability.

Fan et al. found that high heart rate (>100/min) was associated with more symptoms (*p* = 0.047) and a worse performance status (*p* = 0.001) [42]. They suggested that “sustained attention on the change trajectory of vital signs like heart rates and blood pressure of advanced cancer patients is important, which may help make the prediction of patient’s clinical course”.

## 4. Discussion

This scoping review identified a moderate number of studies that investigated the association between abnormal vital signs and prognostication in patients with advanced cancer (i.e., survival of days to weeks). These studies varied in many respects, and most involved limited (often single) assessments of the main vital signs. However, it appears that changes in these vital signs can indicate that a patient is close to death. Nevertheless, some patients maintain “normal” vital signs up to the time of their death [28,45].

As with many aspects of palliative care, further studies are required to clarify the association between abnormal vital signs and prognostication in this cohort of patients. These studies should be adequately powered, prospective in nature, involve outpatients and inpatients, involve serial measurements, and utilise non-invasive methods (wherever possible). Importantly, digital health interventions already exist that allow remote monitoring of certain vital signs [36]. In addition, machine learning/artificial intelligence should be employed to generate novel prognostic models that include vital signs and other relevant prognostic indicators [46].

This scoping review only identified a small number of studies that investigated the generic benefit of measuring vital signs in patients with advanced cancer. Notably, Pavic et al. reported that changes in vital signs preceded unplanned admissions to hospital [36], whilst Kim et al. reported that changes in vital signs preceded “life sustaining treatment” decisions (i.e., decisions to withhold/withdraw applicable interventions) [47]. Hence, measuring vital signs can give an “early warning” of a clinical deterioration, which would facilitate advance care planning [47], and would permit timely treatment, which should limit morbidity (and possibly mortality). Thus, abnormal vital signs may predate symptoms and other signs of the relevant problem (e.g., infection, haemorrhage).

## 5. Conclusions

As previously discussed, vital signs are less frequently utilised in palliative care settings than in secondary care settings. The rationale for this situation is uncertain, especially as many patients receiving palliative care are not in the last days or weeks of life (and so would be appropriate for “active management” of potentially reversible conditions).

However, although the measurement of vital signs may be beneficial to certain patients receiving palliative care, some patients may derive no benefit whatsoever, whilst other patients may be distressed or “harmed” by the processes (especially using conventional methods for measuring vital signs). Hence, the decision to measure vital signs should always be made on a case-by-case basis, and should take into consideration factors such as the patient’s condition (and prognosis), the goals of care, the potential benefits, the potential burdens, and especially the wishes of the patient and their family. Potential burdens include disturbance of the patient (e.g., waking the patient, interference with important social interactions), and physical discomfort (e.g., repositioning the patient, inflation of the blood pressure cuff). It should be noted that the review found no studies that reported such issues, but there were studies involving new, non-invasive methods for measuring vital signs (which could ameliorate some of these issues, and make measuring vital signs more acceptable in this cohort of patients) [34,36,44].

In conclusion, further studies are required to clarify the place of vital sign assessment in specific cohorts of palliative care patients. In the meantime, an individualised approach should be adopted [45].

## Figures and Tables

**Table 1:** Data on vital signs from generic prognostication studies.

Study	Population	Vital Signs Measured	Frequency of Measurement	Significant Findings (Worse Outcome)	Additional Information
Rosenthal et al., 1993 [15]	Hospice × 2 Mixed diagnoses (95% cancer patients) n = 148	Respiratory rate Heart rate Blood pressure Temperature	On admission	Systolic BP <90 mmHg *p* = 0.049 OR = 0.095 [95% CI: 0.009–0.99]	Median survival 14 days
Escalante et al., 2000 [16]	Emergency centre “Cancer patients with acute dyspnea” n = 122	Respiratory rate Heart rate Blood pressure	Time of triage	Multivariate analysis: Respiratory rate >28/min *p* = 0.0000 OR = 12.72 [95% CI: 3.1–52.8] Heart rate ≥110/min or ≤60/min *p* = 0.0025 OR = 4.92 [95% CI: 1.4–16.9]	Endpoint survival < 14 days Respiratory and heart rate were parameters in a model of mortality within 7 days
de Miguel Sanchez et al., 2006 [17]	Home care “Terminally ill cancer patients” n = 98	Respiratory rate Heart rate Temperature	Weekly	Multivariate analysis: Respiratory rate >24/min *p* = 0.005 HR = 2.26 [95% CI: 1.28–4.00] Heart rate >100/min *p* = 0.003 HR = 2.32 [95% CI: 1.33–4.05]	Median survival 32 days
Lam et al., 2007 [18]	Hospital palliative care unit Patients with “advanced cancer” n = 170	Heart rate	Enrolment to study	Univariate analysis *: Heart rate >100/min *p* = 0.009	Median survival 77 days * Heart rate not predictive on multivariate analysis
Chiang et al., 2009 [19]	Hospital palliative care unit “Patients with terminal cancer” n = 324	Respiratory rate Heart rate Temperature	On admission	Multivariate analysis: Respiratory rate ↑ *p* = 0.004 OR = 1.12 [95% CI: 1.04–1.20]	Endpoint survival < 7 days Respiratory rate was a parameter in a model of mortality within 7 days
Kao et al., 2009 [20]	Hospital “Elderly patients with terminal cancer” n = 459	Respiratory rate Heart rate Blood pressure Temperature	On admission (within 24 hr)	Multivariate analysis: Heart rate ↑ *p* = 0.0155 OR = 1.017 [No CI] Systolic BP ↓ *p* = 0.011 OR = 0.985 [No CI]	Endpoint survival < 7 days
Chiang et al., 2010 [21]	Hospital palliative care unit “Patients with terminal cancer” n = 727	Respiratory rate Heart rate Temperature	On admission	Univariate analysis: Respiratory rate ↑ *p* = <0.001 OR = 1.08 [95% CI: 1.04–1.12] Heart rate ↑ *p* = <0.001 OR = 1.02 [95% CI: 1.01–1.03]	Endpoint survival < 7 days Respiratory rate and heart rate were parameters in two computer-assisted models of mortality within 7 days
Elsayem et al., 2010 [22]	Hospital palliative care unit “Patients with advanced cancer” n = 124	Respiratory rate Heart rate Blood pressure Temperature Oxygen saturation	On admission	Multivariate analysis: Respiratory rate ≥21/min *p* = <0.001 OR = 2.15 [95% CI: 1.42–3.26] Heart rate ≥101/min *p* = <0.001 OR = 2.30 [95% CI: 1.44–3.67]	Predictors of inpatient mortality Use of supplemental oxygen was also a predictor of inpatient mortality
Gwilliam et al., 2011 [23]	Palliative care services × 18 “Advanced (locally extensive or metastatic) incurable cancer” n = 1018	Heart rate	Baseline assessment	14-day prediction of survival Heart rate → * p = <0.001 OR = 0.977 [95% CI: 0.965–0.989] 56-day prediction of survival Heart rate → *p* = <0.001 OR = 0.978 [95% CI: 0.967–0.988]	Heart rate was a parameter of the so-called “Prognosis in Palliative care Study PIPS” (predictive model of mortality within 14 days/56 days) * Does not state whether heart rate was high or low
Hwang et al., 2013 [24]	Hospital palliative care unit “Terminally ill cancer patients” n = 181	Heart rate Blood pressure Temperature Oxygen saturation	Not stated (multiple)	Heart rate ↑ (>20%) *p* = 0.01 OR = 0.97 [No CI] PPV = 68.8% Systolic BP ↓ (>20 mmHg)/diastolic BP ↓ (>10 mmHg) *p* = 0.01 OR = 0.96 [No CI] PPV = 78.5% Oxygen saturation <90% *p* = 0.01 OR = 0.96 [No CI] PPV = 81.2%	Endpoint survival < 2 days
Mercadante et al., 2013 [25]	Home care “Patients with advanced cancer” n = 374	Heart rate Blood pressure Temperature	Initial assessment	Multivariate analysis: Heart rate >100/min *p* = 0.005 OR = 3.1 [95% CI: 1.4–6.9] Systolic BP <100 mmHg *p* = 0.002 OR = 2.7 [95% CI: 1.6–5.9]	Endpoint survival < 10 days
Ramchandran et al., 2013 [26]	Hospital “Cancer patients” n = 3062	Respiratory rate Heart rate Blood pressure Temperature Oxygen saturation	On admission (within 24 hr)	Multivariate analysis: Heart rate ↑ *p* = 0.0002 OR = 1.019 [95% CI: 1.01–1.03 Systolic BP ↓ *p* = 0.0024 OR = 0.988 [95% CI: 0.98–1.00] Temperature ↓ *p* = 0.0169 OR = 0.864 [95% CI: 0.77–0.97] Oxygen saturation ↓ *p* = 0.0004 OR = 0.906 [95% CI: 0.86–0.96]	Endpoint survival < 30 days Heart rate, systolic BP, temperature, and oxygen saturation were all parameters in the predictive model of mortality within 30 days
Arai et al., 2014 [27]	Hospital palliative care unit “Patients with terminal cancer” n = 374	Heart rate Blood pressure Body temperature	On admission	Multivariate analysis: Temperature ↓ *p* = 0.05 HR = 0.7 [95% CI: 0.5–1.0]	Endpoint survival < 21 days
Bruera et al., 2014 [28]	Hospital palliative care units × 2 “Patients with advanced cancer” n = 151	Respiratory rate Heart rate Blood pressure Temperature Oxygen saturation	Twice a day	Heart rate ↑ (>10/min) *p* = 0.01 OR = 2.0 [95% CI: 1.1–3.2] Systolic BP ↓ (>20 mmHg) *p* = 0.0004 OR = 2.5 [95% CI: 1.4–4.7] Diastolic BP ↓ (>10 mmHg) *p* = 0.002 OR = 2.3 [95% CI: 1.4–4.3] Temperature ↑ (>0.5 °C) *p* = 0.002 OR 2.1 [95% CI: 1.2–3.9] Oxygen saturation ↓ (>8%) *p* = 0.0003 OR = 3.7 [95% CI: 2.1–10.8]	Endpoint survival < 3 days
Taylor et al., 2014 [29]	Hospital Patients with “solid tumour malignancy” n = 102	Respiratory rate Heart rate Blood pressure Oxygen saturation	Not stated (multiple)	Multilevel modelling: Heart rate ↑ *p* = <0.001 Respiratory rate ↑ *p* = <0.001 Oxygen saturation ↓ *p* = <0.001	Endpoint survival < 14 days
Chen et al., 2015 [30]	Hospital palliative care unit “Patients with advanced cancer” n = 234	Heart rate Blood pressure	On admission	Univariate analysis: Heart rate >120/min *p* = 0.024 OR = 2.10 [95% CI: 1.10–3.40]	Endpoint survival < 7 days Heart rate was a parameter of the so-called “Objective Palliative Prognostic Score” (predictive model of mortality within 7 days)
Chiang et al., 2015 [31]	Hospital palliative care unit “Advanced cancer patients” n = 286	Respiratory rate Heart rate Blood pressure Temperature	On admission	Heart rate ↑ *p* = 0.001 HR = 1.01 [95% CI: 1.01–1.02]	Median survival was 18 days
Sato et al., 2016 [32]	Hospice “Terminal cancer patients” n = 589	Respiratory rate Heart rate Blood pressure Temperature Oxygen saturation	Three times a day	Multivariate analysis: Oxygen saturation ↓ (alert patients) *p* = 0.007 HR = 0.96 [95% CI: 0.93–0.99]	The so-called “Shock index/SI” (heart rate divided by systolic BP) ≥1 was a strong independent risk factor for death
Mori et al., 2020 [33]	Palliative care services “Terminally ill cancer patients with dyspnea at rest” n = 418	Respiratory rate Heart rate	Not stated	Heart rate ↑ *p* = <0.001	Median survival was 13 days
Fukui et al., 2022 [34]	Palliative care unit “Dying cancer patients” n = 24	Respiratory rate Heart rate	Every minute during last 2 weeks of life	Multivariate analysis: Survival < 24 h: Heart rate ↑ *p* = 0.015 OR = 1.024 [95% CI: 1.005–1.043] Survival < 48 h: Respiratory rate ↑ *p* = 0.0084 OR = 1.083 [95% CI: 1.021–1.150] Heart rate ↑ *p* = 0.0005 OR = 1.034 [95% CI: 1.014–1.053] Survival < 72 h: Respiratory rate ↑ *p* = 0.033 OR = 1.100 [95% CI: 1.008–1.120] Heart rate ↑ *p* = 0.001 OR = 1.031 [95% CI: 1.013–1.120]	Similar population to Tanaka et al., 2021
Goh et al., 2022 [40]	Hospital Patients with “advanced cancer” n = 410	Respiratory rate Heart rate Blood pressure Temperature	Baseline assessment	Univariate analysis: Respiratory rate ↑ *p* = <0.0001 OR = 1.1 [95% CI: 1.05–1.16] Heart rate ↑ *p* = 0.0031 OR = 1.01 [95% CI: 1.00–1.02] Systolic BP ↓ *p* = <0.0001 OR = 0.95 [95% CI: 0.94–0.96] Diastolic BP ↓ *p* = <0.0001 OR = 0.95 [95% CI: 0.93–0.96] Temperature ↓ *p* = <0.0019 OR = 0.74 [95% CI: 0.61–0.90]	Endpoint survival < 60 days “Shock index/SI” (heart rate divided by systolic BP) was a strong independent risk factor for death
Aramrat et al., 2023 [41]	Hospital Patients with “cancer and pneumonia” n = 245	Respiratory rate Heart rate Blood pressure Temperature Oxygen saturation	On admission	Multivariate analysis: Oxygen saturation ↓ (<90%) *p* = 0.038 OR = 2.01 [95% CI: 1.04–3.87]	Median survival was 8 days

BP = blood pressure; OR = odds ratio; HR = hazard ratio; RR = relative risk; 95% CI = 95% confidence intervals; PPV = positive predictive value; ↑ = high; ↓ = low.

## Data Availability

The data presented in this study are available in this manuscript.
